# Dissecting the effect of continuous cropping of potato on soil bacterial communities as revealed by high-throughput sequencing

**DOI:** 10.1371/journal.pone.0233356

**Published:** 2020-05-29

**Authors:** Jing Zhao, Dai Zhang, Yiqing Yang, Yang Pan, Dongmei Zhao, Jiehua Zhu, Likui Zhang, Zhihui Yang

**Affiliations:** 1 College of Plant Protection, Agricultural University of Hebei, Baoding City, Hebei Province, China; 2 Technological Innovation Center for Biological Control of Crop Diseases and Insect Pests of Hebei Province, Baoding, China; 3 College of Environmental Science, Yangzhou University, Yangzhou City, Jiangsu Province, China; National Centre For Cell Science, INDIA

## Abstract

Plant rhizosphere-associated bacterial communities play key roles in affecting host health in response to diverse biotic stresses. Currently, the effect of continuous cropping of potato on soil bacterial communities and physiochemical parameters has not been well documented. Herein, we compared bacterial composition and diversity in rotationally and continuously (5, 10, and 30 years) cropped soils, and clarified the correlations between soil properties and the bacterial communities revealed by Illumina MiSeq sequencing. Our results demonstrated that *Proteobacteria*, *Actinobacteria* and *Firmicutes* were the predominant phyla in all the tested soil samples. While the abundance of *Proteobacteria* showed an increase, the abundance of *Actinobacteria* and *Firmicutes* displayed a reduction with the increase of continuous cropping years. At the genus level, as continuous cropping years increasing, the abundance of *Pseudarthrobacter*, *Bacillus* and *Pseudomonas* decreased, but the abundance of *Rhodanobacte*, *Sphingobium*, *Mizugakiibacter* and *Devosia* increased. Our results also demonstrated that the abundance of plant growth-promoting rhizobacteria in the rotationally cropped soil was significantly higher than that of continuously cropped soil. Furthermore, our results showed that soil organic matter, available nitrogen, available phosphorus and available potassium were significantly correlated with bacterial community distribution. Overall, our work provides a comprehensive view of altered structure and composition of bacterial communities between the continuously cropped soil and rotationally cropped soil.

## Introduction

Potato is recognized as the fourth largest staple crop in the world [1] and China is the biggest potato producer worldwide. Potato growing regions in China have increased steadily in recent years. It was estimated that potato growing regions in China exceeded 5.8 million hectares in 2016, thus considerably contributing to agricultural production. With the expansion of planting scale, potato continuous cropping is widespread in China, which caused potato continuous cropping obstacles. Previous studies showed that continuous cropping of potato altered a significant imbalance of soil microbial communities and high frequency of soil-borne diseases, thus leading to sever yield loss of potato [2, 3]. However, the effect of continuous cropping on soil microbial diversity differs from crops. As the increase in continuous cropping years, the bacteria diversity of cotton-planting soil increased firstly and then decreased [4], while the bacterial diversities in cucumber- and tobacco-planting soils decreased [5, 6]. Rotation has been considered as an effective method to alleviate the negative effects of continuous cropping. Furthermore, previous studies showed that rotation can also affect soil bacterial community [7–9]. For example, rotation is able to significantly increase the abundance and diversity of soil microbial communities [10], and increasing plant varieties during crop rotation are capable of enriching the function of agro-ecosystem [11].

Bacteria are highly abundant and widely distributed in soil. During niche colonization, they interact with plants by utilizing the rhizosphere exudates as nutrient sources, affecting plant growth [12]. The soil-borne pathogens, such as *Erwinia*, lead to diseases by growing in plant roots and acquiring nutrients from the plant roots [13]. However, some bacteria can maintain plant health by either secreting beneficial compounds or controlling the action or growth of plant pathogens [14–18]. *Enterobacter* and *Rahnella* produce indole compounds that can promote plant growth [16]. *Pseudomonas spp*. secretes several types of antibiotics that can inhibit the growth of pathogenic bacteria [19] and *Bacillus spp*. serves as a bio-control agent to suppress soil-borne microorganisms [20].

The balance of bacterial communities is helpful for plant growth [21]. Bacterial communities would be affected by several factors, among which crop species and soil physical-chemical properties are thought to be the most important ones. Furthermore, pH, organic matter, potassium, nitrogen and phosphorus content among chemical properties considerably affect bacterial communities [22, 23]. Soil bacteria can play essential roles in maintaining soil environment by improving the resistance of soil to external changes [24]. Therefore, it would be important to gain insights into the environmental factors affecting soil bacterial communities for exploring soil microecosystems.

The effect of continuous cropping of potato on soil microbial communities was revealed by various methods. By using dilution-plate method, continuous cropping of potato reduced the abundance of bacterial communities in soil [25]. The reduced diversity of soil bacterial communities measured by using BIOLOG ECO technology was observed in continuous cropping of potato [26]. Furthermore, Min et al. (2017) showed that continuous cropping of potato reduce the diversity of bacterial communities and the abundance of beneficial bacteria in soil by employing T-RFLP technique [27]. These findings suggest that continuous cropping of potato is capable of reducing the abundance and diversity of rhizosphere bacteria. However, the approaches employed in these experiments are unable to identify non-cultured microorganisms present in the soil, which brings a barrier to comprehensively understand soil bacterial communities in rhizosphere of continuous cropping of potato.

Compared with conventional molecular biology approaches, the next-generation sequencing technology can be employed to probe compositions of soil microbial communities, which overcomes the inability to detect non-cultured and rare microorganisms in the soil. By using Illumina sequencing technology, microbial compositions of rhizosphere associated with different plant species such as cherries, rice, and tobacco, have been investigated [28]. In this study, we employed the advanced Illumina MiSeq platform to sequence the 16S rDNA and determined the rhizosphere soil bacterial communities in continuous cropping and rotation field of potato, respectively. The soil bacterial communities, the effect of cropping patterns on bacterial community structure, and the correlation between bacterial community structure and environmental factors were discussed in this work.

## Materials and methods

### Ethics statement

We state clearly that no specific permissions were required for these field studies because the land is owned by the country, and relevant national laws allow researchers to take samples for scientific research. Our samples were all taken under the consent and companionship of the land growers. No specific permission is required, and they are fully complied with Chinese laws. Furthermore, our samples were all taken from potato farmland. We confirm that the field studies did not involve endangered or protected species.

### Soil sample collection

The field experimental sites were located at Weichang, Chengde, Hebei Province (41°2'44''N, 117°57'14'' E) in Northern China. The annual average temperature and sunshine duration were in the range of 0.50~6.00°C and 2577~2832 hours, respectively. About 58~64% of sunshine occurred per year. Annual precipitation was 300~560 mm. Inorganic fertilizer was applied during potato planting. All the soil samples were taken from the rhizosphere of the potato at flowering stage field in 2017, and the soil types were brown soil. Continuous cropping soils were collected from the field where only potato was planted for 5 years (S-F-5), 10 years (S-F-10), and 30 years (S-F-30), respectively. On the other hand, rotation soil (R-F) was taken from the field with 5-year history of alternating planting of potato and corn. By using a five-point (W) sampling method, five soil samples from five sites were collected from each cropping potato field, and then the five samples were mixed as one treatment, this process was repeated three times as three repetitions of each treatment. The soil samples were treated by removing loose soil from potato roots and putting attached soils into sterile ziplock bags [29], placed in the 4°C incubator and then transferred to the laboratory. Next, the soil samples were sieved (mesh size 1×1 mm^2^) and then placed into sterile 50ml centrifuge tubes and stored at -80°C freezer for determining soil physic-chemical properties and bacterial communities.

### Measurement of soil physic-chemical properties

The organic matter (OM) was measured using the potassium dichromate volumetric method-outside heating method [30]. The determination of available nitrogen (AN) was performed using the alkali solution diffusion method [31]. Determination of available phosphorus (AP) was conducted using 0.5 mol/L sodium bicarbonate method [32]. Available potassium (AK) was measured using ammonium acetate extraction flame photometry method [33]. Water soluble calcium (Ca^2+^) and magnesium (Mg^2+^) were measured using atomic absorption spectrophotometry [34]. The soil pH was measured by pH meter.

### DNA extraction and sequencing

Total DNA was extracted from soil samples collected from each cropping potato field with the TIANamp Soil DNA Kit (Tiangen biotech, Beijing). By using the extracted DNA as template, the V3-V4 region of 16s DNA was amplified in the presence with a pair of universal primers (347F: CCT ACG GRR BGC ASC AGK VRV GAA T, and 802R: GGA CTA CNV GGG TWT CTA ATC C). For sequencing library preparations, the obtained PCR products were added Index linker using PCR. Library quality was measured using the Agilent 2100 Bioanalyzer (Agilent Technologies, Palo Alto, CA, USA) [35] and library concentration was measured by a Qubit 2.0 Fluorometer (Invitrogen, Carlsbad, Calif.) [36]. Library was sequenced using an Illumina MiSeq with paired-end 300 bp reads with MiSeq Control Software (MCS) (Illumina, San Diego, CA, USA) [37].

### Operational taxonomic unit (OTU) picking and statistical analyses

The forward and reverse reads generated from sequencing was firstly merged and low-quality filtered, then the chimera sequences were removed to obtain the effective sequences. After obtaining the effective sequences, sequences were grouped into operational taxonomic units (OTUs) using the clustering program VSEARCH (1.9.6) against the Silva 119 database pre-clustered at 97% sequence identity [38]. The Ribosomal Database Program (RDP) classifier was used to assign taxonomic category to all OTUs at a confidence threshold of 0.8. The RDP classifier utilizes the Silva 123 database [39] that has taxonomic categories predicted to the species level. Sequences were filtered prior to calculation of alpha and beta diversity statistics [40]. Alpha diversity indexes were calculated in QIIME from rarefied samples using the Shannon index for diversity, using the Chao1 index for richness. Beta diversity was calculated using weighted and unweighted UniFrac. Redundancy analysis (RDA) was calculated to show the relationship between bacterial communities and environment factors. All statistical analyses were performed using IBM SPSS Statistics 19. All sequences were submitted to the NCBI Sequence Read Archive (SRA) with the accession number (PRJNA549054).

## Results

### Soil physicochemical properties

To investigate the effect of soil environmental factors on soil bacterial communities, we examined the soil pH, OM, AN, AP, AK, Ca^2+^ and Mg^2+^ (Table 1). Our results showed that the pH values slightly fluctuated in different treatments, ranging from 7.16 for S-F-30 to 7.53 for R-F. The contents of OM, AN and AP exhibited the same trend in different soils, where the highest content was observed in S-F-10, followed by S-F-30, S-F-5, and R-F. In addition, S-F-10 had the highest content of AK at 307.22 mg/kg, followed by S-F-5 at 284.60 mg/kg, and R-F contained the lowest content at 103.60 mg/kg. Furthermore, the content of Ca^2+^ in S-F-5 was the highest at 290.40 mg/kg, followed by S-F-30 at 263.07 mg/kg. By contrast, the content of Ca^2+^ in S-F-10 and R-F were estimated to be only 32.68 mg/kg and 13.69 mg/kg, respectively, demonstrating that the contents of Ca^2+^ in S-F-10 and R-F were significantly lower than those in S-F-5 and S-F-30. Last, the content of Mg^2+^ varied from the soil samples, following the order of S-F-30 at 44.98 mg/kg, S-F-5 at 40.68 mg/kg, S-F-10 at 26.60 mg/kg and R-F at 12.90 mg/kg. Thus, significant fluctuations occurred in soil environmental factors between treatments, and these fluctuations might alter the diversity of soil bacterial communities.

**Table 1:** Physical and chemical properties of the soil samples.

Sample	pH	OM (g/kg)	AN (mg/kg)	AP (mg/kg)	AK (mg/kg)	Ca^2+^ (mg/kg)	Mg^2+^ (mg/kg)
R-F	7.53±0.03A	12.05±0.44D	48.57±1.23C	44.19±049D	103.60±0.00D	13.69±0.18B	12.90±0.31D
S-F-5	7.20±0.02C	17.66±0.24C	68.69±0.93B	69.45±0.08C	284.60±1.51B	290.40±19.31A	40.68±0.83B
S-F-10	7.36±0.02B	25.07±0.32A	94.99±1.61A	96.41±0.49A	307.22±0.00A	32.68±1.99B	26.60±0.74C
S-F-30	7.16±0.02C	19.73±0.81B	72.98±0.93B	83.92±0.32B	249.79±0.00C	263.07±5.39A	44.98±0.33A

Values are presented as the mean ± standard deviation (n = 3). Different letters in the same column indicate a significant difference at P < 0.01. OM: organic matter, AN: available nitrogen, AP: available phosphorus, AK: available potassium, Ca^2+^: water-soluble calcium, Mg^2+^: water-soluble magnesium. R-F: rotation soil; S-F-5: potato continuous cropping for 5 years; S-F-10: potato continuous cropping for 10 years; S-F-30: potato continuous cropping for 30 years.

### Sequencing data

**Fig 1:**
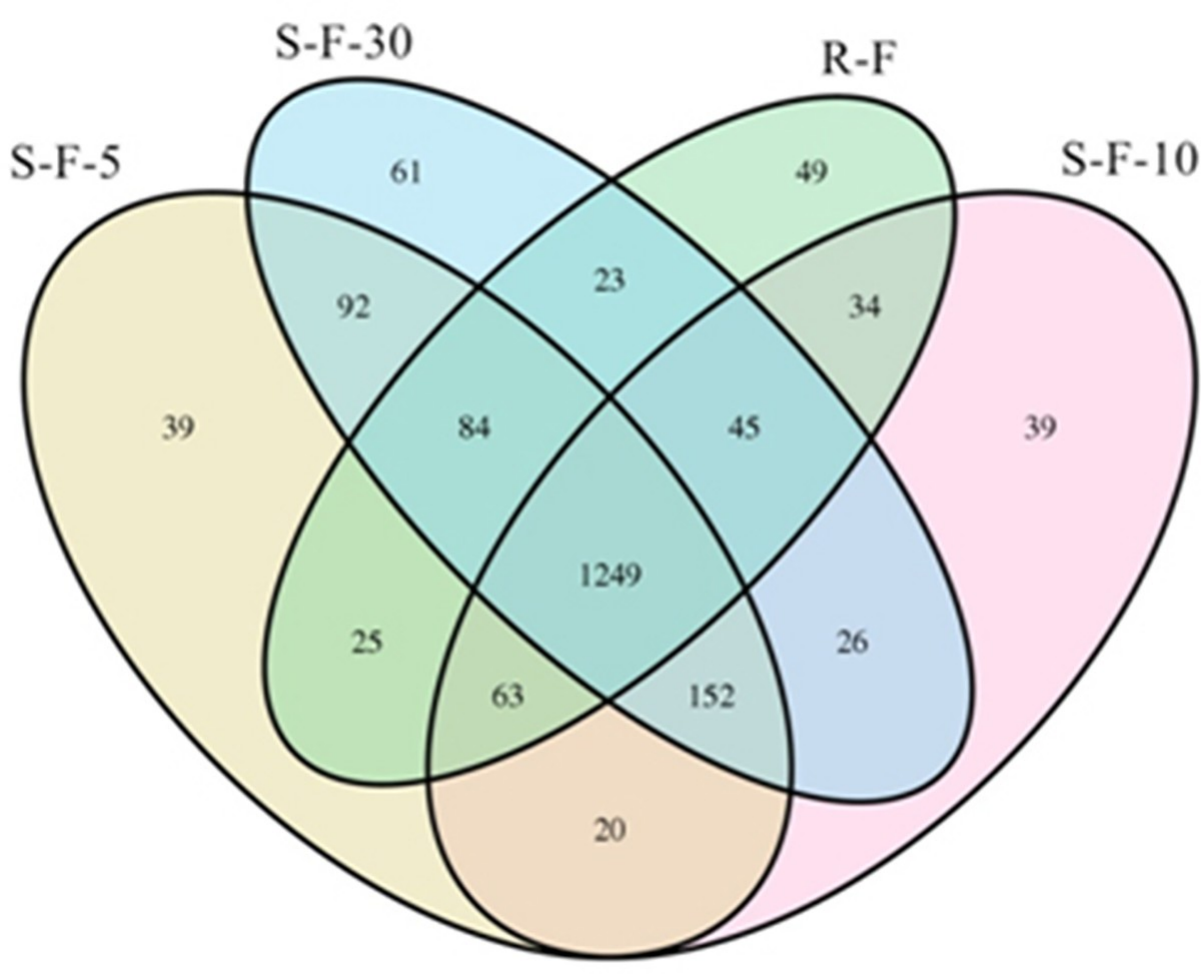
A Venn diagram of the OTUs for bacterial communities in each soil sample. Shared and unique OTUs in each sample were estimated at 97% similarity. The numbers of OTUs are indicated inside the diagrams. R-F: rotation soil; S-F-5: soil of potato continuous cropping for 5 years; S-F-10: soil of potato continuous cropping for 10 years; S-F-30: soil of potato continuous cropping for 30 years.

The Illumina-based analysis of the hypervariable V3-V4 region of the bacterial 16S rRNA gene produced 1,608,854 total reads. After filtering and removing potential erroneous sequences, a total of 804,427 effective reads were obtained. According to 97% similarity, a total of 2,001 OTUs for bacterial diversity were generated from the four samples, and the order of the OTU numbers ranging from high to low was 1732 for S-F-30, 1724 for S-F-5, 1628 for S-F-10 and 1572 for R-F (Table 2). A Venn diagram was created, which showed that the four samples shared 1,249 OTUs (Fig 1), suggesting the presence of similar bacterial groups in each sample. Furthermore, various numbers of shared and unique OTUs exist in the four samples, suggesting that the four soils have different bacterial community structure, albeit harboring similar bacterial species.

**Table 2:** Diversity index of bacterial communities of the soil samples.

Sample	OTU (97%)	ACE	Chao1	Shannon	Coverage
R-F	1572	1459.38±26.50C	1493.19±25.62C	8.28±0.06D	0.99±0.00A
S-F-5	1724	1596.24±24.95A	1616.84±29.27AB	8.45±0.02C	0.99±0.00A
S-F-10	1628	1528.86±18.40B	1553.50±7.09BC	8.62±0.02B	0.99±0.00A
S-F-30	1732	1625.01±17.85A	1657.91±36.60A	8.77±0.03A	0.99±0.00A

Values are presented as the mean ± standard deviation (n = 3). Different letters in the same column indicate a significant difference at P < 0.01. R-F: rotation soil; S-F-5: soil of potato continuous cropping for 5 years; S-F-10: soil of potato continuous cropping for 10 years; S-F-30: soil of potato continuous cropping for 30 years.

α-diversity index of bacterial communities was estimated at the significance level of 0.01, including ACE, Chao 1 and Shannon. As shown in Table 2, the index of ACE, Chao 1 and Shannon in S-F-5, S-F-10, and S-F-30 samples were significantly higher than those in R-F sample. Furthermore, the coverage percentages for all the four samples were estimated to be 99%, thus suggesting that only few sequences were not detected. Thus, α-diversity index of bacterial communities showed that the numbers of OTU, bacterial species and diversity in the continuously cropping were significantly higher than those in rotation cropping. Specifically, the bacterial diversity increased as the increase in the continuously cropping years, and the S-F-30 sample harbored maximum bacterial diversity. All rarefaction curves approached an asymptote (S1 Fig), indicating that the sampling depth was sufficient to capture the whole bacterial communities in each sample.

### Bacterial community analysis

**Fig 2:**
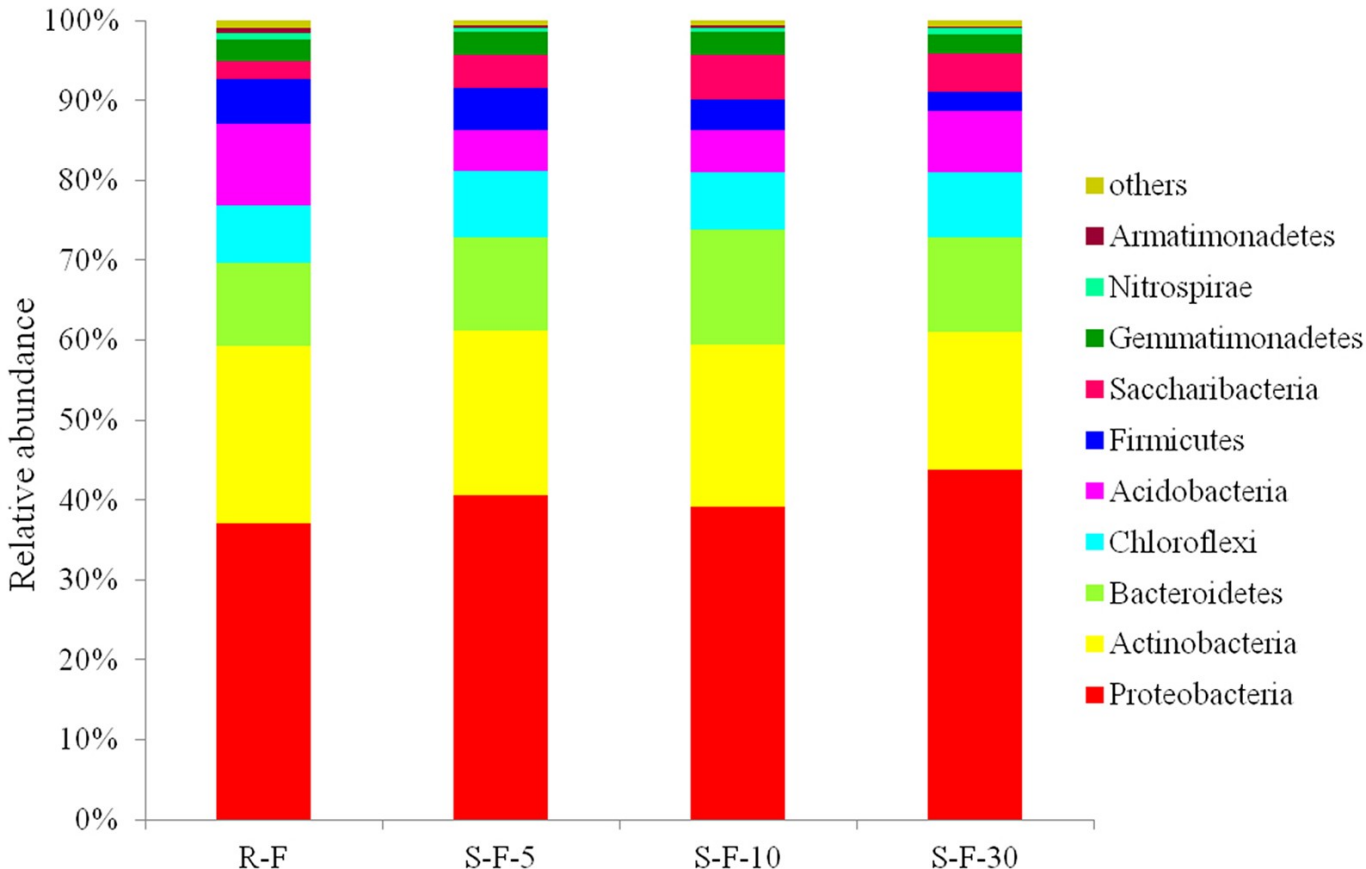
Relative abundance of bacterial communities at 10 most abundant phylum levels in each soil sample. Each color represents the percentage of each phylum in each sample. The abscissa represents different treatments, and the ordinate represents percentage of phylum. R-F: rotation soil; S-F-5: soil of potato continuous cropping for 5 years; S-F-10: soil of potato continuous cropping for 10 years; S-F-30: soil of potato continuous cropping for 30 years.

At the phylum level, a total of 386,015 effective reads were created in all the four samples, covering 32 phyla. Among them, 23 phyla were identified in R-F sample, 22 in S-F-5, 23 in S-F-10, and 26 in S-F-30. As shown in Fig 2 and S1 Table, the relative abundance of 8 phyla, including *Proteobacteria*, *Actinobacteria*, *Bacteroidetes*, *Chloroflexi*, *Acidobacteria*, *Firmicutes*, *Saccharibacteria* and *Gemmatimonadetes*, was more than 1%. The relative abundance of *Proteobacteria* was the highest in all the tested samples. Interestingly, the relative abundance of *Proteobacteria* of S-F-5, S-F-10 and S-F-30 samples was higher than that of R-F sample, and the S-F-30 samples harbored the highest abundance.

*Actinobacteria* was the second dominant phylum in the tested samples. The R-F sample possessed 22.31%, which was higher than that of S-F-5, S-F-10 and S-F-30 samples. Furthermore, the relative abundance of *Actinobacteria* in the continuously cropping decreased as the increase in planting years.

Furthermore, the S-F-5, S-F-10 and S-F-30 samples had higher relative abundance of *Bacteroidetes* and *Saccharibacteria* than that of the R-F sample. S-F-10 sample had the highest abundance of *Bacteroidetes* and *Saccharibacteria*. *Chloroflexi* was found in R-F sample with a relative abundance of 7.15%, which was lower than all the three continuous cropping samples. However, S-F-5 sample had the highest abundance of *Chloroflexi*.

In addition, the R-F sample had higher relative abundance of *Actinobacteria* and *Firmicutes* than the three continuous cropping samples. The relative abundance of *Actinobacteria* and *Firmicutes* decreased as extending the planting years. Last, a reduced abundance of *Gemmatimonadetes* was observed in S-F-5, S-F-10 and S-F-30 samples with the increase of cropping years. While the S-F-5, S-F-10 samples had higher abundance of *Gemmatimonadetes* than R-F sample, the R-F sample had higher abundance of *Gemmatimonadetes* than S-F-30 sample.

**Fig 3:**
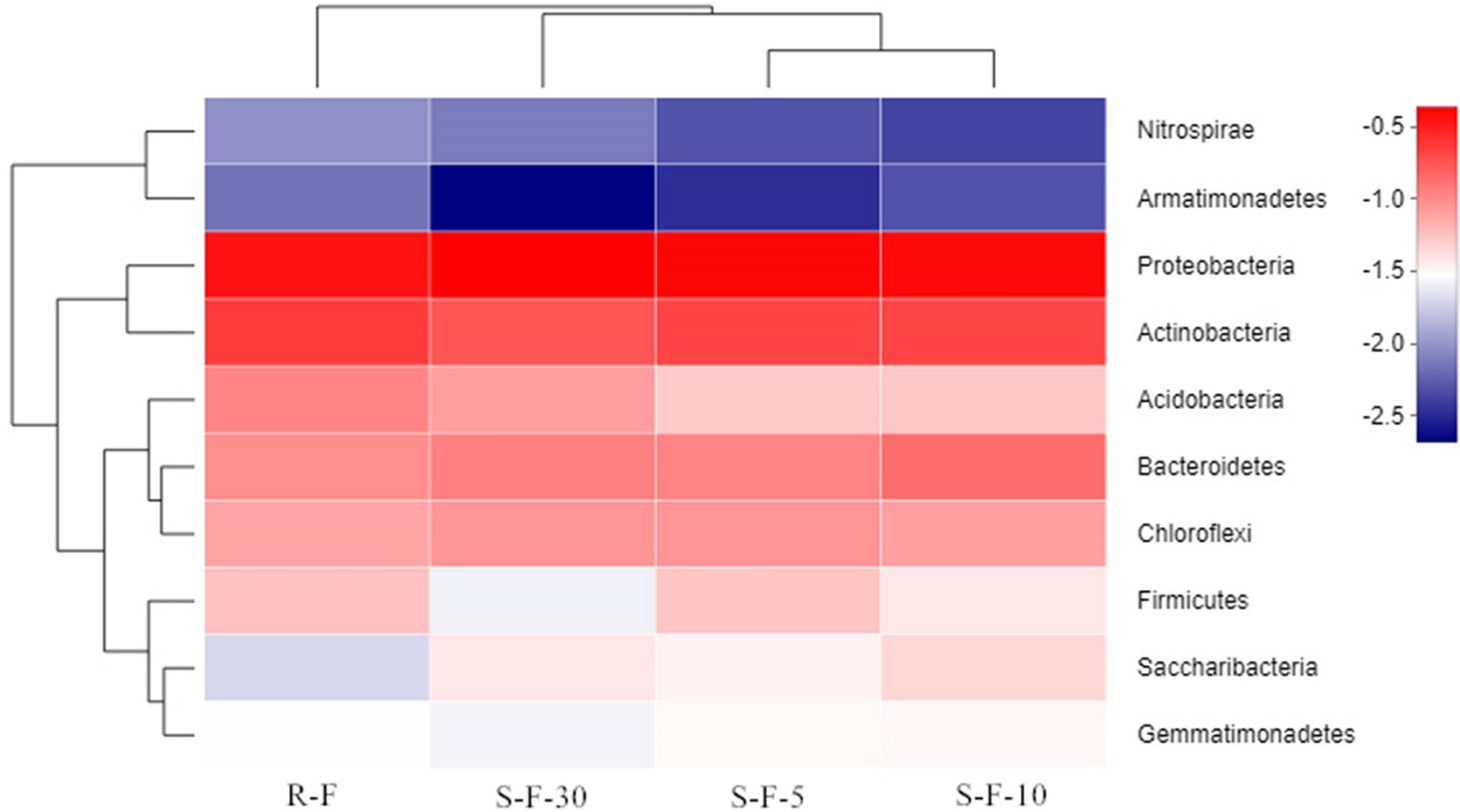
Hierarchical clustering heat-map of bacterial communities at 10 most abundant phylum levels in each soil sample. The phylogenetic tree was constructed by the neighbor-joining method. The relative percentage of each phylum within each soil sample (vertical clustering) or among four samples (horizontal clustering) are shown in the heat-map. The relative values for each phylum was indicated with color intensity at the right of the figure. R-F: rotation soil; S-F-5: soil of potato continuous cropping for 5 years; S-F-10: soil of potato continuous cropping for 10 years; S-F-30: soil of potato continuous cropping for 30 years.

**Fig 4:**
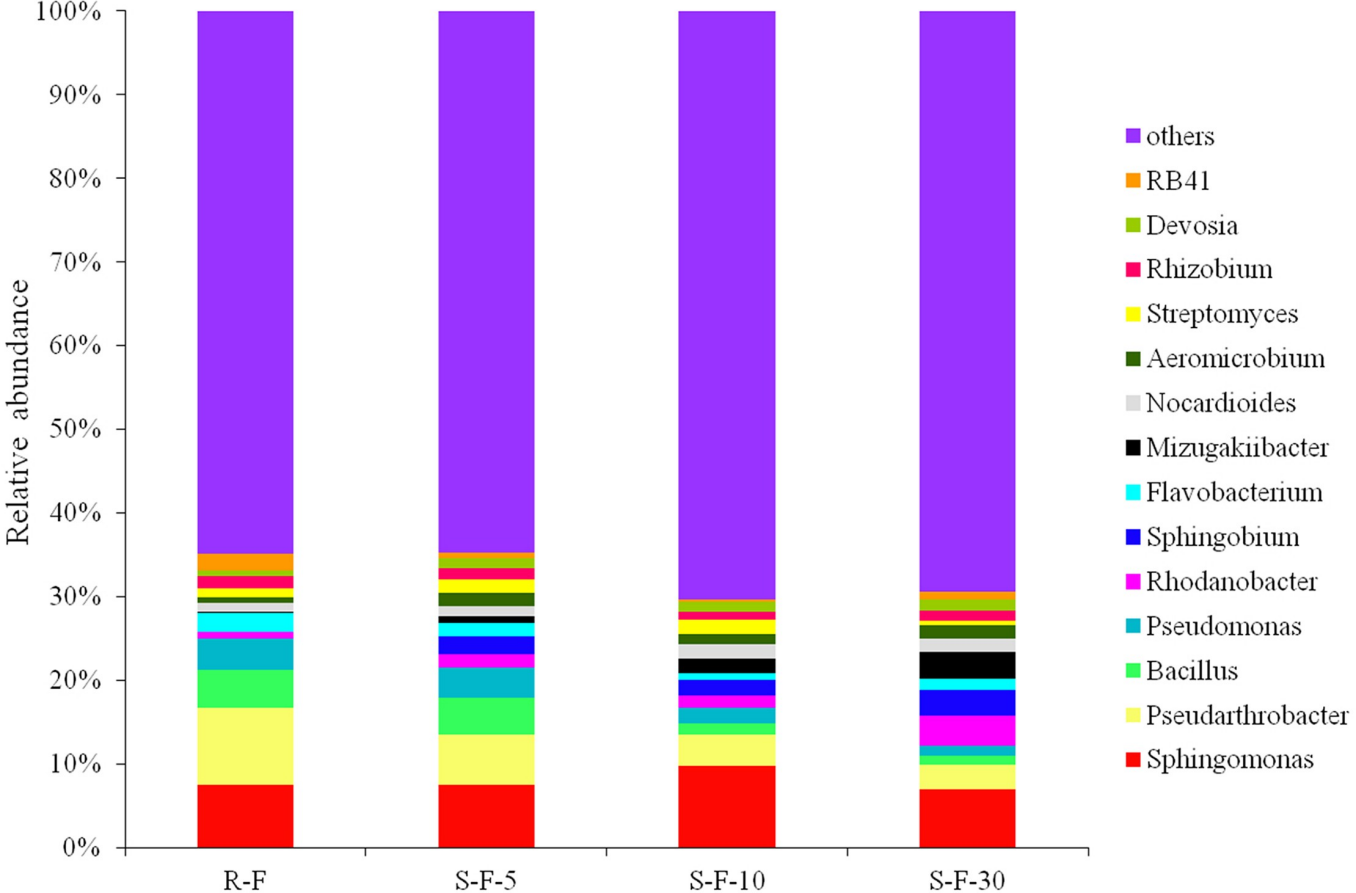
Relative abundances of bacterial communities at 14 most abundant genus levels in each soil sample. Each color represents the percentage of each genus in each sample. The abscissa represents different treatments, and the ordinate represents percentage of genera. R-F: rotation soil; S-F-5: soil of potato continuous cropping for 5 years; S-F-10: soil of potato continuous cropping for 10 years; S-F-30: soil of potato continuous cropping for 30 years.

**Fig 5:**
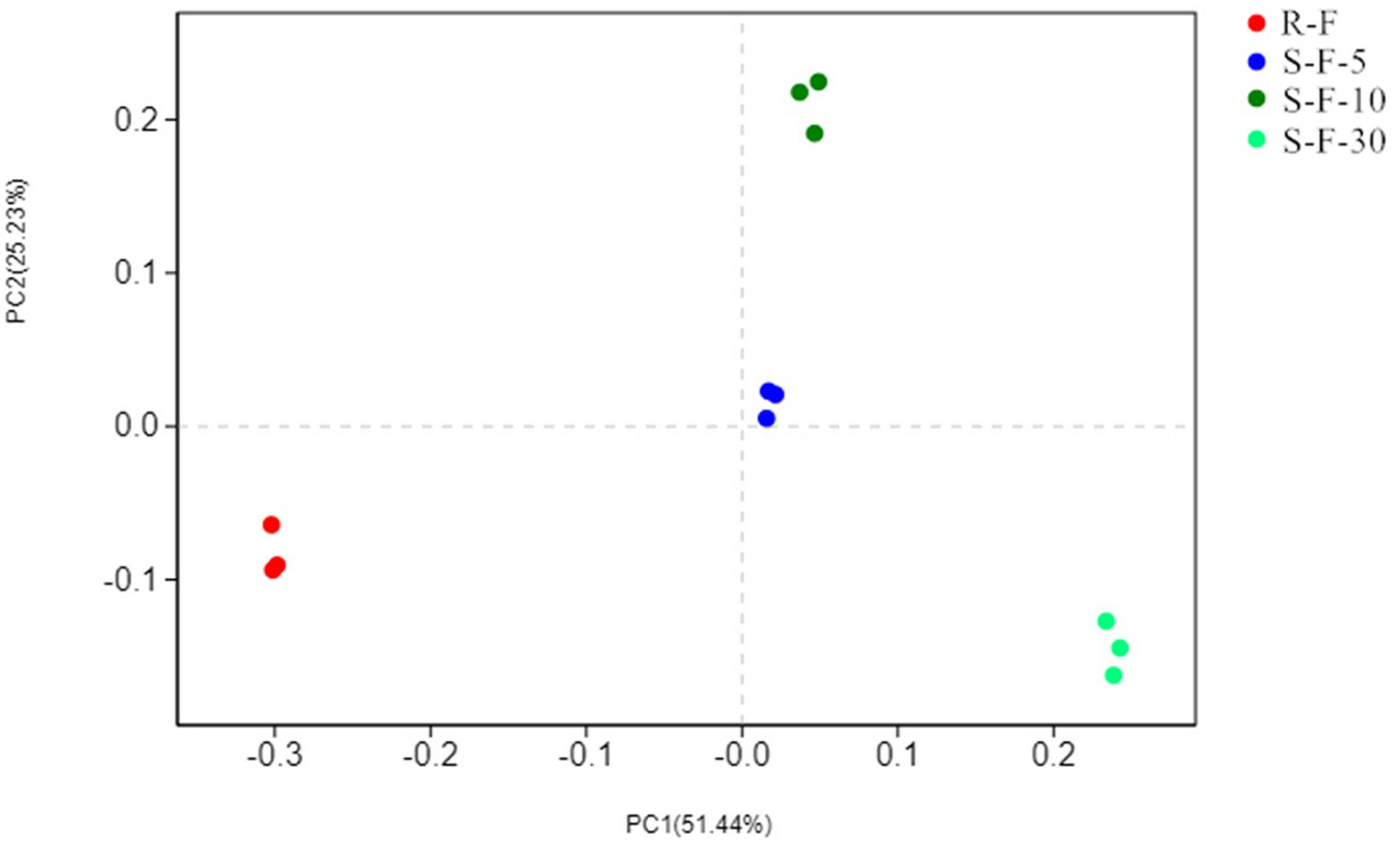
Principal coordinates analysis of OTUs in each soil sample. Percent variability explained by each principal component is shown in parentheses after each axis legend. R-F: rotation soil; S-F-5: soil of potato continuous cropping for 5 years; S-F-10: soil of potato continuous cropping for 10 years; S-F-30: soil of potato continuous cropping for 30 years.

The 10 most abundant phyla in all four samples were analyzed by hierarchically clustering heat mapping to reveal microbial similarity. As shown in Fig 3, S-F-5 and S-F-10 samples were clustered into one branch in four soil samples, and the rotated soil samples were separated from the three continuous samples. These observations suggest that S-F-5 and S-F-10 samples had the highest similarity among the three continuous cropping samples, and the difference between R-F and the three continuous cropping samples was greater than the difference between the three continuous cropping samples.

At the genus level, 260 genera were identified in all the tested soil samples: 221 genera in the R-F sample, 247 genera in S-F-5 sample, 232 genera in S-F-10 sample, and 235 genera in S-F-30 sample. As shown in Fig 4 and S2 Table, the tested samples harbored 14 genera with a relative abundance of higher than 1%. The R-F sample harbored the higher relative abundance of *Pseudarthrobacter*, *Bacillus* and *Pseudomonas* than the three continuous cropping samples, and a reduced relative abundance was found in the three continuous cropping samples as increasing cropping years. By contrast, the R-F sample had the lower relative abundance of *Rhodanobacte*, *Sphingobium*, *Mizugakiibacter* and *Devosia* than the three continuous cropping samples, and an elevated relative abundance was observed in the three continuous cropping samples as extending cropping years.

Furthermore, the R-F sample displayed higher relative abundance of *Flavobacterium*, *Rhizobium* and RB41 than S-F-5, S-F-10 and S-F-30 samples. However, *Nocardioides*, *Aeromicrobium* and *Rhodanobacter* were identified in the R-F sample with a relative abundance lower than S-F-5, S-F-10 and S-F-30 samples.

At the species level, 32 species were identified in all the tested soil samples. As shown in S3 Table, the tested samples harbored 11 species with a relative abundance of higher than 0.1%. The relative abundances of *Bacillus aryabhattai*, *Bacillus drentensis*, *Bacillus simplex* and *Rhizobium etli* decreased as extending of the planting years. However, the relative abundance of *Rhodococcus erythropolis* increased as the increase in planting years. In addition, the relative abundances of *Paenarthrobacter nitroguajacolicus*, *Acinetobacter calcoaceticus*, *Rhizobium etli* and *Sphingobacterium multivorum* in rotation soils were significantly higher than those in continuous cropping soils.

### Bacterial community structure

The Principal coordinates analysis (PCoA) based on the Bray-Curtis matrix algorithm could clearly reflect the differences in soil microbial communities between different samples. The first principal component of PCoA explained 51.44% of the total variation in bacterial communities, and the second principal component explained 25.23% of the variation in bacterial communities (Fig 5). In continuous cropping and rotation samples, the three replicates of each treatment were clustered together and displayed good repeatability. The rotation sample and the three continuous samples were well separated on the first principal component, and the difference between the three continuous samples was mainly expressed on the second principal component. Among them, the sample with the highest similarity to R-F was S-F-5, while S-F-5 and S-F-10 showed the highest similarity. However, the similarity between S-F-30 and S-F-10 is low with S-F-5, which may be due to the high similarity between S-F-5 and S-F-30 environmental factors.

### Relationship between bacterial community structure and soil properties

**Fig 6:**
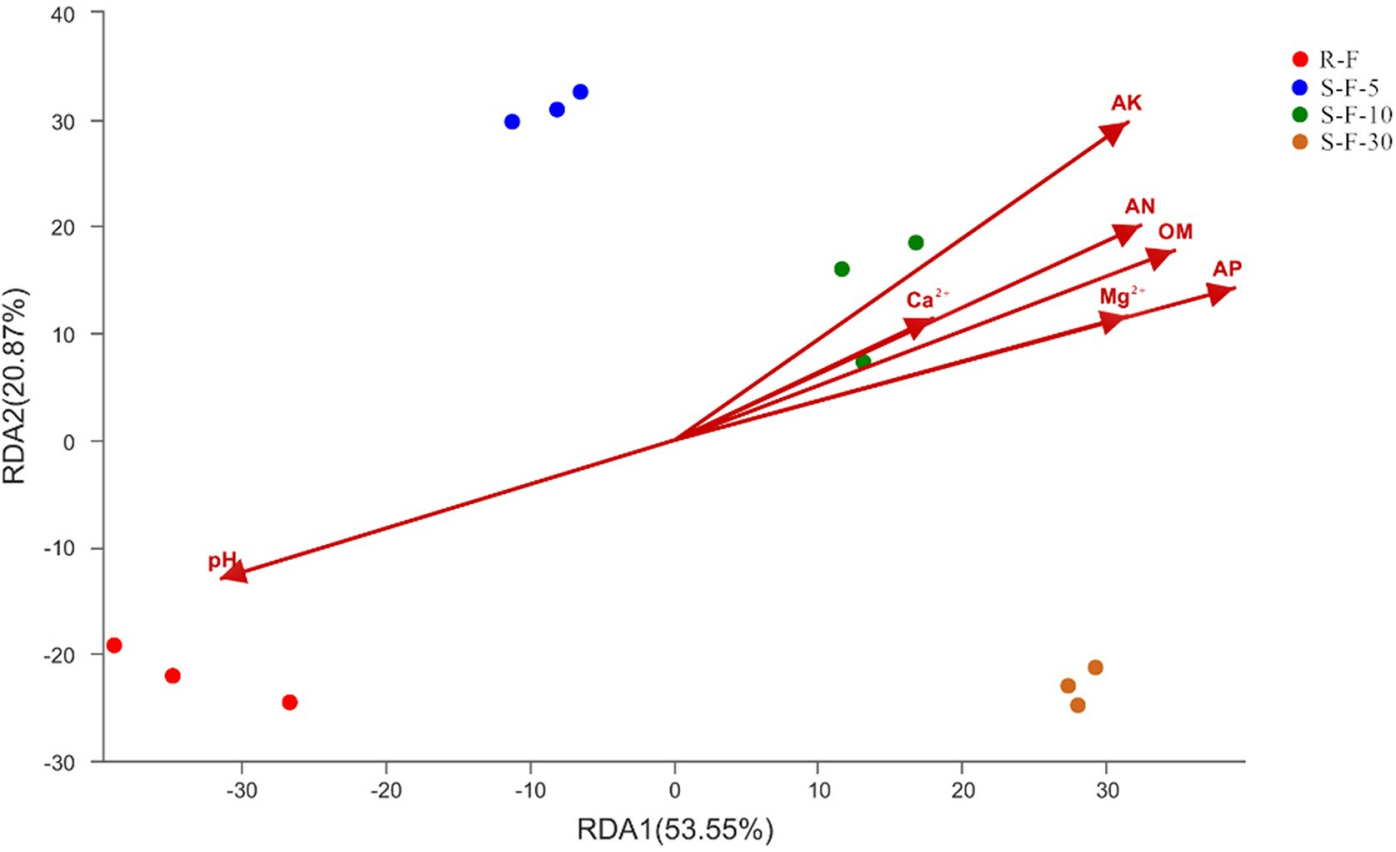
Redundancy analysis (RDA) between the environmental factors and the bacterial communities. Percent variability explained by each principal component is shown in parentheses after each axis legend. AN, available nitrogen; AP, available phosphorus; AK, available potassium; OM, organic matter; Ca^2+^, water-soluble calcium; Mg^2+^, water-soluble magnesium. R-F: rotation soil; S-F-5: soil of potato continuous cropping for 5 years; S-F-10: soil of potato continuous cropping for 10 years; S-F-30: soil of potato continuous cropping for 30 years.

Redundancy analysis (RDA) clearly shows the effects of different environmental factors on soil microbial communities and explains the correlation between environmental factors and soil microbes. The first two axes of RDA showed 53.55% and 20.87% of the total variation, respectively, and the total variation of the two axes was 74.42% (Fig 6). Among them, the microbial communities of continuous cropping and rotation showed a large difference in spatial distribution. Besides, the soil bacterial community under rotation was strongly correlated with each physical and chemical property. In addition, except for pH, the effects of different environmental factors (AK, AN, Ca^2+^, OM, AP and Mg^2+^) on soil bacterial communities were positively correlated. At the significant level of 0.01, among all the environmental factors, the most significant effect on the bacterial communities of rhizosphere soil was AK, the correlation coefficient was 0.001, followed by OM and AP, the correlation coefficient was 0.002, and finally AN, correlation coefficient was 0.007. Ca^2+^, Mg^2+^ and pH showed a low correlation with soil bacterial communities, and the correlation coefficients were 0.308, 0.031 and 0.018, respectively (Table 3). Overall, we concluded that the rotation cropping displays more sensitive effect on the correlation between soil bacterial communities and soil environmental factors than continuous cropping; AK, OM, AP and AN were key environmental factors for affecting bacterial communities of soil.

**Table 3:** Significance of the soil physicochemical properties in explaining the bacteria community structure obtained from the RDA results.

Property	R^2^	P values
OM	0.7958	0.002
AN	0.7602	0.007
AP	0.8977	0.002
AK	0.9847	0.001
pH	0.6090	0.018
Ca^2+^	0.2361	0.308
Mg^2+^	0.5885	0.031

AN, available nitrogen; AP, available phosphorus; AK, available potassium; OM, organic matter; Ca^2+^, water-soluble calcium; Mg^2+^, water-soluble magnesium.

**Fig 7:**
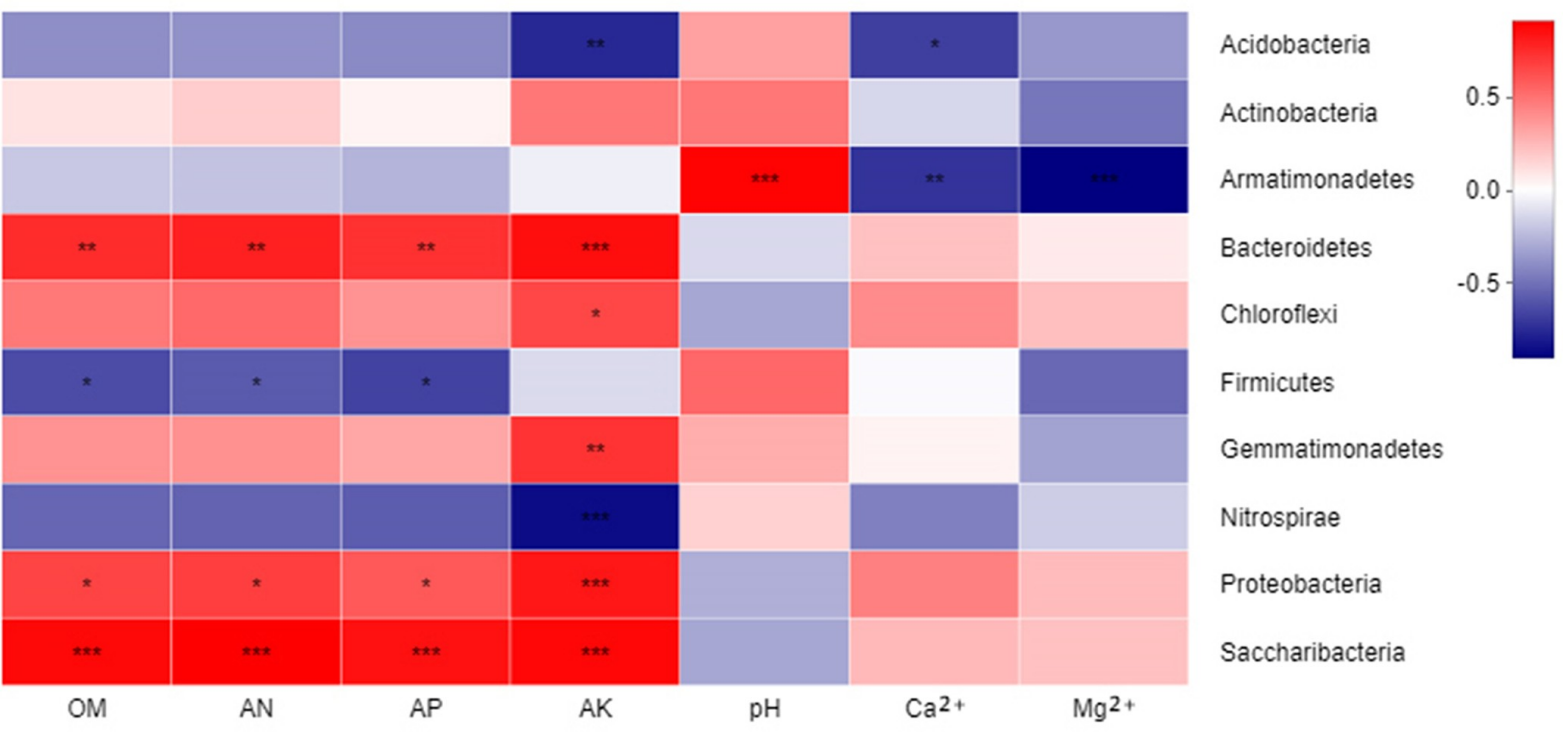
A correlation heatmap between environmental factors and bacterial communities at the phylum level. Correlations between environmental factors and phylum are indicated by different colors and "*". AN, available nitrogen; AP, available phosphorus; AK, available potassium; OM, organic matter; Ca^2+^, water-soluble calcium; Mg^2+^, water-soluble magnesium. R-F: rotation soil; S-F-5: soil of potato continuous cropping for 5 years; S-F-10: soil of potato continuous cropping for 10 years; S-F-30: soil of potato continuous cropping for 30 years.

Furthermore, a correlation heatmap between environmental factors and bacterial communities at the phylum level was constructed (Fig 7). At a significant level of 0.01, *Proteobacteria* displayed significantly positively correlation with AK. A similarly significant positive correlation between the bacteria *Bacteroidetes* and *Saccharibacteria* and environmental factors OM, AN, AP and AK were revealed. By contrast, *Acidobacteria* was negatively correlated with AK, and *Gemmatimonadetes* was positively correlated with AK.

## Discussion

In this work, we used a barcoded Illumina high-throughput sequencing platform to investigate soil bacterial communities in the continuous cropping and rotation of potato, compared difference of bacterial communities between the continuous cropping and rotation soils, and revealed the correlation between and bacterial communities and soil properties. 10 phyla common to all samples were detected at higher abundance, which most abundant were *Proteobacteria*, *Actinobacteria*, *Bacteroidetes*, *Chloroflexi*, *Acidobacteria*, and *Firmicutes*. Furthermore, other 22 phyla were also detected at lower abundance. Overall, the rhizosphere soils for the continuous cropping and rotation of potato harbor abundant bacterial communities.

Diversity of soil bacterial communities is largely affected by soil environmental factors [41]. Previous studies showed that soil OM is a key factor affecting the diversity of soil microbial communities [42]. High content of soil OM can be helpful for increasing the diversity of soil bacterial communities [43]. Soil nitrogen is thought to promote the decomposition of OM by soil microorganisms and increase the diversity of bacterial community [44]. Furthermore, phosphorus and potassium in the soil are also thought to increase the diversity of soil bacterial communities [43, 45]. In our study, the Shannon indexes of soil bacterial communities generally increased with the increase of the OM, AN, and AP contents in the soils, which is consistent with previous results [42, 45]. Although the contents of OM, AN, AP and AK in S-F-10 were the highest, the diversity of soil bacterial communities was lower than that in S-F-30. This may be due to the low contents of Ca^2+^ and Mg^2+^ in S-F-10, especially Ca^2+^. The Ca^2+^ content in S-F-10 was only 32.68 mg/kg, which was much lower than that in S-F-5 and S-F-30. Alloway suggested that calcium and magnesium are essential elements of the organisms [46], thereby playing a vital role in keeping the health and productivity of the soil ecosystem, which are conducive to the maintenance of nutrients and the attachment of microorganisms. Thus, the reduced content of Ca^2+^ and Mg^2+^ in S-F-10 might be the main reason for the abnormal Shannon index of the bacterial communities in S-F-10. Overall, balanced nutrients are of great significance for maintaining the diversity of soil bacterial communities.

Our results also revealed that continuous cropping can reduce the abundance of beneficial bacteria in soil (Fig 2). The abundance of beneficial bacteria *Actinobacteria* and *Firmicutes* decreased with the time of continuous cropping, which is consistent to the previous observation on vanilla as described by Xiong et al (2015) [47]. Previous studies suggest that the abundance of *Actinobacteria* and *Firmicutes* is positively correlated with the crop health [48] and *Actinobacteria* is one of the key factors causing bacteriostatic effect on soil [13].

Furthermore, our studies indicated that the abundance of bio-control bacteria *Bacillus aryabhattai*, *Bacillus drentensis* and *Bacillus simplex* decreased with the increase of continuous cropping years (S3 Table). These *Bacillus* species can effectively inhibit the growth of the pathogens such as *Rhizoctoniasolani* and *Botrytiscinerea* by secreting fengycin, bacillaene, difficidin and iturins, and they can also antagonize pathogens through nutrient competition [49, 50]. Thus, the reduced abundance of beneficial bacteria in continuous cropping in our work suggest that continuous cropping is a key obstacle to maintain relative abundances of beneficial bacteria in soil for promoting plant growth and resistance to biotic stresses.

Our results demonstrated that rotation significantly changed the bacterial community structure in soil. Compared with continuous cropping field soil in the rhizosphere of potato rotation field, the abundances of *Paenarthrobacter nitroguajacolicus*, *Acinetobacter calcoaceticus* and *Rhizobium etli* were higher than those in the continuous cropping soils (S3 Table). Many studies indicate that these species are the plant growth promoting rhizobacteria (PGPR) in soil. Wakako *et al* (2013) suggested that *A*. *calcoaceticus* can promote the growth of plant [51]. *P*. *nitroguajacolicus* not only can enhance plant growth, but also have the ability to enhance the release of jasmonic acid from plant [52]. Besides, *R*. *etli* can induce systemic resistance of plants to *Globodera pallida*. Thus, the PGPR, which is relatively abundant in soil, can protect plants by inhibiting the occurrence of soil-borne diseases. In this study, a significant difference was observed in potato rhizosphere bacterial community structure between the continuous cropping field soil and rotation field soil at the flowering stage (S3 Table), which indicates that rotation can maintain the health of soil bacterial communities.

Besides, Our results demonstrated that bacterial communities in the rotation and continuous cropping soils are predominantly affected by AK, OM, AP and AN, which is consistent with the findings that the soil bacterial communities were significantly affected by soil SOC, AK, AN, AP and pH [53]. By contrast, previous studies showed that soil pH to be the strongest factor shaping microbial community structure [54, 55], which appears inconsistent with our results. This may be due to the mild fluctuations of soil pH (varied from 7.53 to 7.16 in all samples) that would slightly alter bacterial communities in the soil samples.

## Conclusion

In this work, we investigated bacterial diversity in rhizosphere soils of continuous cropping and rotation of potato by high-throughput sequencing, demonstrating that *Proteobacteria*, *Actinobacteria* and *Firmicutes* were abundant in all the soil samples. Our results also revealed change of bacterial community structure with the continuously cropping years and the correlation between bacterial communities and soil properties. In future work, we will try to investigate more about the effect of long-term continuous cropping on soil bacterial communities and potato growth.

## Supporting information

S1 Fig (DOCX)

S1 Table (DOCX)

S2 Table (DOCX)

S3 Table (DOCX)

## References

[1] Goo Y, Kim T, Lee M, Lee S. Accumulation of PrLeg, a perilla legumin protein in potato tuber results in enhanced level of sulphur-containing amino acids. Cr Biol. 2013;336(9):433–439. 10.1016/j.crvi.2013.09.002 24161240

[2] Quan S, Ru C, Nai-ping S, Qing-feng W, Rui W. Change trends of soil nutrients, enzyme activities, and microbial composition in continuous potato cropping system in semi-arid and cool hilly area of Ningxia. J Soil Water Conserv. 2010;24(6):208–212. 10.1080/00949651003724790

[3] Qin S, Yeboah S, Wang D, Zhang J. Effects of ridge-furrow and plastic mulching planting patterns on microflora and potato tuber yield in continuous cropping soil. Soil Use Manage. 2016;32(3):465–473. 10.1111/sum.12291

[4] Wei Z, Yu D. Analysis of the succession of structure of the bacteria community in soil from long-term continuous cotton cropping in Xinjiang using high-throughput sequencing. Arch Microbiol. 2018;200(4):653–662. 10.1007/s00203-018-1476-4 29352369

[5] Yao H, Jiao X, Wu F. Effects of continuous cucumber cropping and alternative rotations under protected cultivation on soil microbial community diversity. Plant Soil. 2006;284(1–2):195–203. 10.2307/24123922

[6] She S, Niu J, Zhang C, Xiao Y, Chen W, Dai L, et al Significant relationship between soil bacterial community structure and incidence of bacterial wilt disease under continuous cropping system. Arch Microbiol. 2017;199(2):267–275. 10.1007/s00203-016-1301-x 27699437

[7] Bailey KL, Lazarovits G. Suppressing soil-borne diseases with residue management and organic amendments. Soil Till Res. 2003;72(2):169–180. 10.1016/S0167-1987(03)00086-2

[8] Curl EA, Truelove B. The Rhizosphere, Springer-Verlag; 1986.

[9] O'Donnell AG, Seasman M, Macrae A, Waite I, Davies JT. Plants and fertilizers as drivers of change in microbial community structure and function in soil. Plant Soil. 2011;232(1/2):135–145. 10.1023/A:1010394221729

[10] Venter ZS, Jacobs K, Hawkins H. The impact of crop rotation on soil microbial diversity: A meta-analysis. Pedobiologia. 2016;59(4):215–223. 10.1016/j.pedobi.2016.04.001

[11] Smith RG, Gross KL, Robertson GP. Effects of crop diversity on agroecosystem function: crop yield response. Ecosystems. 2008;11(3):355–366. 10.2307/40296291

[12] Bulgarelli D, Schlaeppi K, Spaepen S, van Themaat EVL, Schulze-Lefert P. Structure and functions of the bacterial microbiota of plants. Annu Rev Plant Biol. 2013 2013-01-20;64(1):807–38. 10.1146/annurev-arplant-050312-120106 23373698

[13] Mendes R, Kruijt M, de Bruijn I, Dekkers E, van der Voort M, Schneider JHM, et al Deciphering the rhizosphere microbiome for disease-suppressive bacteria. Science. 2011;332(6033):1097–1100. 10.1126/science.1203980 21551032

[14] Fabra A, Castro S, Taurian T, Angelini J, Ibañez F. Interaction among *Arachis hypogaea* L. (peanut) and beneficial soil microorganisms: how much is it known? Crit Rev Microbiol. 2010;36(3):179–194. 10.3109/10408410903584863 20214416

[15] Raaijmakers JM, Mazzola AM. Diversity and natural functions of antibiotics produced by beneficial and plant pathogenic bacteria. Annu Rev Phytopathol. 2012;50(50):403–424. 10.1146/annurev-phyto-081211-172908 22681451

[16] Costa PBD, Granada CE, Ambrosini A, Moreira F, de Souza R, Dos Passos JFM, et al A model to explain plant growth promotion traits: A multivariate analysis of 2,211 bacterial isolates. Plos One. 2014;9(12):e116020 10.1371/journal.pone.0116020 25542031 PMC4277451

[17] Mingna C, Xiao L, Qingli Y, Xiaoyuan C, Lijuan P, Na C, et al Dynamic succession of soil bacterial community during continuous cropping of peanut (*Arachis hypogaea* L.). Plos One. 2014;9(7):e101335 10.1371/journal.pone.0101355 25010658 PMC4092034

[18] Souza RD, Ambrosini A, Passaglia LMP. Plant growth-promoting bacteria as inoculants in agricultural soils. Genet Mol Biol. 2015;38(4):401–419. 10.1590/S1415-475738420150053 26537605 PMC4763327

[19] Ligon J, Hill D, Hammer P, Torkewitz N, Hofmann D. Natural products with antifungal activity from *Pseudomonas* biocontrol bacteria. Pest Manag Sci. 2015;56(8):688–695. 10.1002/1526-4998(200008)56:83.0.CO;2-V

[20] Jamil S, Hui T, Mingshan J. *Bacillus* species as versatile weapons for plant pathogens: a review. Biotechnol Biotec Eq. 2017;31(3):446–459. 10.1080/13102818.2017.1286950

[21] Jun Z, Ruifu Z, Chao X, Weibing X, Li S, Yangchun X, et al Pyrosequencing reveals contrasting soil bacterial diversity and community structure of two main winter wheat cropping systems in China. Microb Ecol. 2014;67(2):443–453. 10.1007/s00248-013-0322-0 24276539

[22] Fierer N, Jackson RB. The diversity and biogeography of soil bacterial communities. PANS. 2006;103(3):626–631. 10.1073/pnas.0507535103 16407148 PMC1334650

[23] Sun L, Gao J, Huang T, Kendall JRA, Shen Q, Zhang R. Parental material and cultivation determine soil bacterial community structure and fertility. Fems Microbiol Ecol. 2014;91(1):1–10. 10.1093/femsec/fiu010 25764534

[24] Girvan M, Campbell C, Killham K, Prosser J, LA Glover. Bacterial diversity promotes community stability and functional resilience after perturbation. Environ Microbiol. 2005;7(3):301–313. 10.1111/j.1462-2920.2004.00695.x 15683391

[25] De Zhen M, Xiao DG, Xue LT, Tian WG. Effect of potato continuous cropping on rhizosphere soil microbial physiological colony. Chin J Gansu Agr Uni. 2016;51(02):35–39. 10.13432/j.cnki.jgsau.2016.02.007

[26] Du Q, Di L, Kun M. Effect of potato continuous cropping on soil microbial community structure and function. Chin J Ecol Env Sci. 2012;21(07):1252–1256. 10.16258/j.cnki.1674-5906.2012.07.016

[27] Min QX, Yi Z, Li T, Qiang LG. Crop rhizospheric microbial community structure and functional diversity as affected by maize and potato intercropping. J Plant Nutr. 2017;40(17):2402–2412. 10.1080/01904167.2017.1346674

[28] Xie W, Su J, Zhu Y. Phyllosphere bacterial community of floating macrophytes in paddy soil environments as revealed by Illumina High-Throughput Sequencing. Appl Environ Microb. 2015;81(2):522–532. 10.1128/AEM.03191-14 25362067 PMC4277568

[29] Smalla K, Cresswell N, Mendonca-Hagler LC, Wolters A, Elsas JDV. Rapid DNA extraction protocol from soil for polymerase chain reaction-mediated amplification. J App Bacteriol. 1993;74(1):78–85. 10.1111/j.1365-2672.1993.tb02999.x

[30] Kun PL. Evaluation of uncertainty of soil organic matter measured by potassium dichromate outside heating method. Chin J Constr Des Eng. 2017;(16):101–102. 10.13616/j.cnki.gcjsysj.2017.08.146

[31] Xiang-Sheng Y. Comparison of soil available nitrogen concentration between flow injection method and alkali-diffusion method. Chin J Anhui Agr Sci. 2011;39(20):12166–12167. 10.13989/j.cnki.0517-6611.2011.20.170

[32] LiuK Q Z, S. T. Processing tomato phosphorus utilization and post-harvest soil profile. Can J Soil Sci. 2017;91(3):417–425. 10.4141/CJSS09098

[33] Cheng J, Ding C, Li X, Zhang T, Wang X. Soil quality evaluation for navel orange production systems in central subtropical China. Soil Till Res. 2016;115:225–232. 10.1016/j.still.2015.08.015

[34] Macphee WSG, Ball ADF. Routine determination of calcium and magnesium in soil extracts by atomic absorption spectrophotometry. J Sci Food Agr. 2010;18(8):376–380. 10.1002/jsfa.2740180812

[35] Eric G, Odilo M, Andreas P. Semiquantitative reverse transcription-polymerase chain reaction with the Agilent 2100 Bioanalyzer. Electrophoresis. 2012;22(18):4016–4022. 10.1002/1522-2683(200110)22:18<4016::aid-elps4016>3.0.co;2-9 11700735

[36] Xiaolei L, Weiguo H, Hailiang D, Shang W, Hongchen J, Geng W, et al Distribution and diversity of *Cyanobacteria* and eukaryotic algae in Qinghai-Tibetan lakes. Geomicrobiol J. 2016;23(10):860–869. 10.1080/01490451.2015.1120368

[37] Caporaso JG, Lauber CL, Walters WA, Berg-Lyons D, Huntley J, Fierer N, et al Ultra-high-throughput microbial community analysis on the Illumina HiSeq and MiSeq platforms. ISME J. 2012;6(8):1621–1624. 10.1038/ismej.2012.8 22402401 PMC3400413

[38] Patrick DS, Handelsman J. Introducing DOTUR, a computer program for defining operational taxonomic units and estimating species richness. Appl Environ Microb. 2005;71(3):1501–1506. 10.1128/AEM.71.3.1501-1506.2005 PMC1065144 15746353

[39] Quast C, Pruesse E, Yilmaz P, Gerken J, Schweer T, Yarza P, et al The SILVA ribosomal RNA gene database project: improved data processing and web-based tools. Nucleic Acids Res. 2012;41(D1):D590–D596. 10.1093/nar/gks1219 23193283 PMC3531112

[40] Jost L. Partitioning diversity into independent alpha and beta components. Ecology. 2007;88(10):2427–2439. 10.1890/06-1736.1 18027744

[41] Torsvik V, Rheim RS, Yr JG. Total bacterial diversity in soil and sediment communities-A review. J Ind Microb. 1996;17(3–4):170–178. 10.1007/bf01574690

[42] Schnürer J, Clarholm M, Rosswall T. Microbial biomass and activity in an agricultural soil with different organic matter contents. Soil Biol Biochem. 1985;17(5):611–618. 10.1016/0038-0717(85)90036-7

[43] Ge Y, Zhang J, Zhang L, Yang M, He J. Long-term fertilization regimes affect bacterial community structure and diversity of an agricultural soil in northern China. J Soil Sediment. 2008;8(1):43–50. 10.1065/jss2008.01.270

[44] Cusack DF, Silver WL, Torn MS, Burton SD, Firestone MK. Changes in microbial community characteristics and soil organic matter with nitrogen additions in two tropical forests. Ecology. 2011;92(3):621–632. 10.1890/10-0459.1 21608471

[45] Yunfu G, Xiaoping Z, Shihua T, Kristaina L. Soil microbial biomass, crop yields, and bacterial community structure as affected by long-term fertilizer treatments under wheat-rice cropping. Eur J Soil Biol. 2009;45(3):239–246. 10.1016/j.ejsobi.2009.02.005

[46] Alloway BJ. Bioavailability of elements in soil. Essent Med Geol. 2013:351–373. 10.1007/978-94-007-4375-5_15

[47] Xiong W, Li Z, Liu H, Xue C, Zhang R, Wu H, et al The effect of long-term continuous cropping of black pepper on soil bacterial communities as determined by 454 pyrosequencing. Plos One. 2015;10(8):e0136946 10.1371/journal.pone.0136946 26317364 PMC4552827

[48] Li R, Khafipour E, Krause DO, Entz MH, de Kievit TR, Fernando WG. Pyrosequencing reveals the influence of organic and conventional farming systems on bacterial communities. Plos One. 2012;7(12):e51897 10.1371/journal.pone.0051897 23284808 PMC3526490

[49] Ongena M, Jacques P. *Bacillus* lipopeptides: versatile weapons for plant disease biocontrol. Trends Microbiol. 2008;16(3):115–125. 10.1016/j.tim.2007.12.009 18289856

[50] Jeong H, Jeong D, Kim SH, Song GC, Park S, Ryu C, et al Draft genome sequence of the plant growth-promoting bacterium *Bacillus siamensis* KCTC 13613T. J Bacteriol. 2012;194(15):4148–4149. 10.1128/JB.00805-12 22815459 PMC3416560

[51] Suzuki W, Sugawara M, Miwa K, Morikawa M. Plant growth-promoting bacterium *Acinetobacter calcoaceticus* P23 increases the chlorophyll content of the monocot Lemna minor (duckweed) and the dicot Lactuca sativa (lettuce). J Biosci Bioeng. 2013;118(1):41–44. 10.1016/j.jbiosc.2013.12.007 24468072

[52] Santhanam R, Baldwin IT, Groten K. In wild tobacco, Nicotiana attenuata, variation among bacterial communities of isogenic plants is mainly shaped by the local soil microbiota independently of the plants' capacity to produce jasmonic acid. Comm Int Biol. 2015;8(2):e1017160 10.1080/19420889.2015.1017160 26478769 PMC4594342

[53] Li YC, Li Z, Li ZW, Jiang YH, Weng BQ, Lin WX. Variations of rhizosphere bacterial communities in tea (*Camellia sinensis* L.) continuous cropping soil by high-throughput pyrosequencing approach. J Appl Microbiol. 2016;121(3):787–799. 10.1111/jam.13225 27377624

[54] Rousk J, Bååth E, Brookes PC, Lauber CL, Lozupone C, Caporaso JG, et al Soil bacterial and fungal communities across a pH gradient in an arable soil. ISME J. 2010;4(10):1340–1351. 10.1038/ismej.2010.58 20445636

[55] Shen C, Xiong J, Zhang H, Feng Y, Lin X, Li X, et al Soil pH drives the spatial distribution of bacterial communities along elevation on Changbai Mountain. Soil Biol Biochem. 2013;57:204–211. 10.1016/j.soilbio.2012.07.013

