# Supplementary material for: Dissecting the effect of continuous cropping of potato on soil bacterial communities as revealed by high-throughput sequencing
Source: PLoS One. 2020 May 29;15(5):e0233356. doi: 10.1371/journal.pone.0233356 (PMC7259506; doi:10.1371/journal.pone.0233356)
Supplement: S1 Fig — (DOCX) [file pone.0233356.s001.docx]

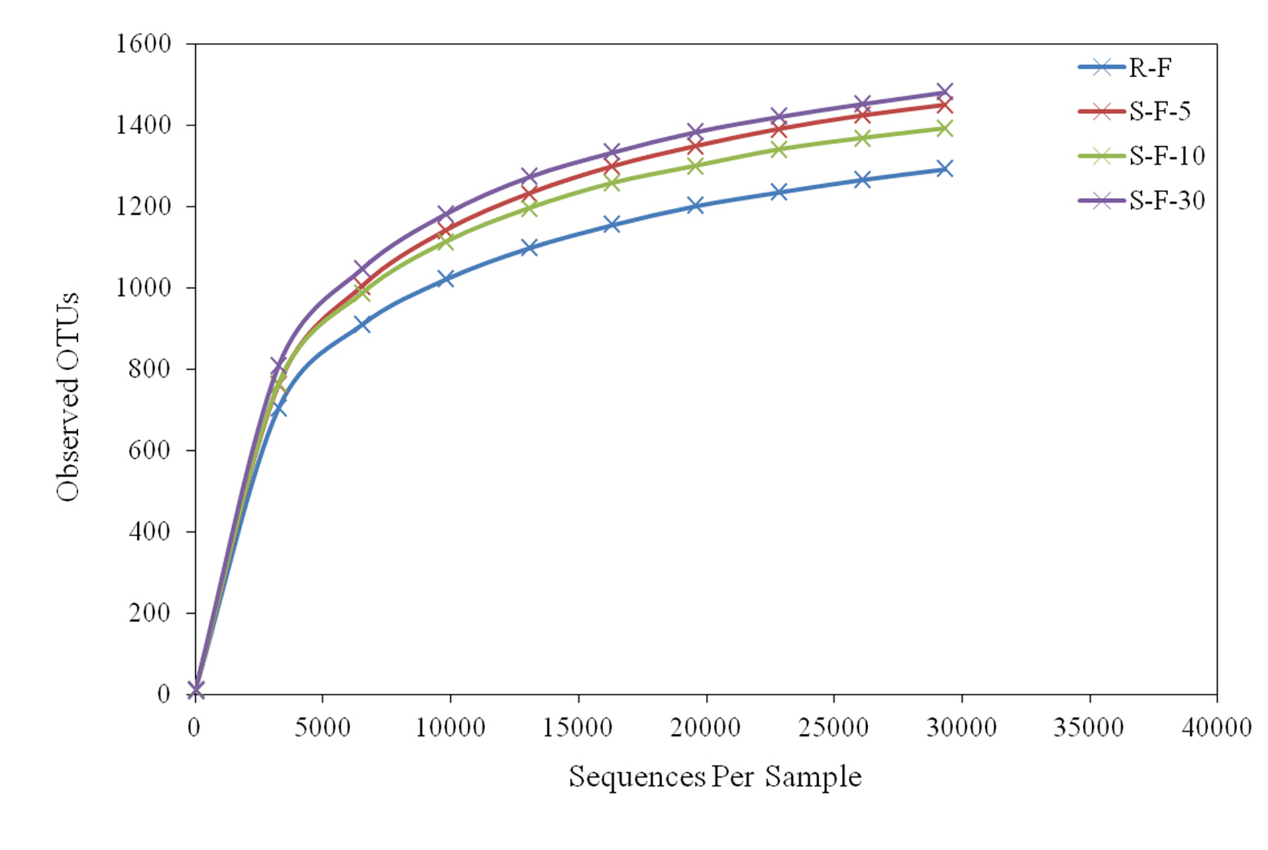
Fig. S1

**Fig. S1.** Rarefaction curves of 16S rDNA sequences of bacterial communities in each soil sample. Rarefaction curves were generated based on OTUs at 97% similarity. R-F: rotation soil; S-F-5: soil of potato continuous cropping for 5 years; S-F-10: soil of potato continuous cropping for 10 years; S-F-30: soil of potato continuous cropping for 30 years.
