# Supplementary material for: Dissecting the effect of continuous cropping of potato on soil bacterial communities as revealed by high-throughput sequencing
Source: PLoS One. 2020 May 29;15(5):e0233356. doi: 10.1371/journal.pone.0233356 (PMC7259506; doi:10.1371/journal.pone.0233356)
Supplement: S1 Table — (DOCX) [file pone.0233356.s002.docx]

**Table S1 Relative abundance of phyla with Percentage >1%.**

| Phyla | R-F (%) | S-F-5 (%) | S-F-10 (%) | S-F-30 (%) |
| --- | --- | --- | --- | --- |
| *Proteobacteria* | 36.99±0.90C | 40.64±0.68B | 39.11±1.24B | 43.73±0.34A |
| *Actinobacteria* | 22.31±1.73A | 20.50±1.20AB | 20.35±1.92AB | 17.33±0.99B |
| *Bacteroidetes* | 10.43±0.69B | 11.72±1.22B | 14.36±0.99A | 11.81±0.65B |
| *Chloroflexi* | 7.15±0.14A | 8.34±1.01A | 7.23±0.62A | 8.15±0.40A |
| *Acidobacteria* | 10.12±0.69A | 5.04±0.31C | 5.24±0.09C | 7.65±0.44B |
| *Firmicutes* | 5.66±0.40A | 5.24±0.56A | 3.88±0.26B | 2.37±0.14C |
| *Saccharibacteria* | 2.22±0.24C | 4.15±0.18B | 5.58±0.26A | 4.73±0.23B |
| *Gemmatimonadetes* | 2.72±0.10BC | 3.01±0.08A | 2.86±0.13AB | 2.52±0.09C |

Values are presented as the mean ± standard deviation (n=3). Different letters in the same column indicate a significant difference at p < 0.01. R-F: rotation soil; S-F-5: soil of potato continuous cropping for 5 years; S-F-10: soil of potato continuous cropping for 10 years; S-F-30: soil of potato continuous cropping for 30 years.
