# Supplementary material for: Dissecting the effect of continuous cropping of potato on soil bacterial communities as revealed by high-throughput sequencing
Source: PLoS One. 2020 May 29;15(5):e0233356. doi: 10.1371/journal.pone.0233356 (PMC7259506; doi:10.1371/journal.pone.0233356)
Supplement: S2 Table — (DOCX) [file pone.0233356.s003.docx]

**Table S2 Relative abundance of genera with Percentage >1%.**

| Genera | R-F (%) | S-F-5 (%) | S-F-10 (%) | S-F-30 (%) |
| --- | --- | --- | --- | --- |
| *Sphingomonas* | 7.45±0.11B | 7.46±0.29B | 9.77±0.17A | 6.99±0.27B |
| *Pseudarthrobacter* | 9.29±0.92A | 6.09±0.38B | 3.74±0.51C | 2.93±0.23C |
| *Bacillus* | 4.56±0.32A | 4.32±0.41A | 1.38±0.08B | 1.00±0.13B |
| *Pseudomonas* | 3.73±0.61A | 3.68±0.21A | 1.87±0.28B | 1.24±0.13B |
| *Rhodanobacter* | 0.73±0.16C | 1.61±0.17B | 1.39±0.05B | 3.64±0.28A |
| *Sphingobium* | 0.06±0.01C | 2.02±0.05B | 1.87±0.17B | 3.07±0.18A |
| *Flavobacterium* | 2.18±0.11A | 1.64±0.67AB | 0.87±0.14B | 1.30±0.01AB |
| *Mizugakiibacter* | 0.16±0.04D | 0.87±0.06C | 1.68±0.19B | 3.23±0.06A |
| *Nocardioides* | 1.10±0.11C | 1.15±0.14BC | 1.73±0.18A | 1.54±0.17AB |
| *Aeromicrobium* | 0.65±0.07B | 1.54±0.23A | 1.28±0.08A | 1.58±0.12A |
| *Streptomyces* | 1.10±0.02B | 1.63±0.06A | 1.62±0.15A | 0.63±0.03C |
| *Rhizobium* | 1.45±0.11A | 1.39±0.17AB | 1.01±0.10B | 1.08±0.18AB |
| *Devosia* | 0.63±0.04C | 1.15±0.05B | 1.14±0.06B | 1.34±0.09A |
| *RB41* | 2.09±0.28A | 0.66±0.09BC | 0.37±0.04C | 1.00±0.04B |

Values are presented as the mean ± standard deviation (n=3). Different letters in the same column indicate a significant difference at p < 0.01. R-F: rotation soil; S-F-5: soil of potato continuous cropping for 5 years; S-F-10: soil of potato continuous cropping for 10 years; S-F-30: soil of potato continuous cropping for 30 years.
