# Supplementary material for: Dissecting the effect of continuous cropping of potato on soil bacterial communities as revealed by high-throughput sequencing
Source: PLoS One. 2020 May 29;15(5):e0233356. doi: 10.1371/journal.pone.0233356 (PMC7259506; doi:10.1371/journal.pone.0233356)
Supplement: S3 Table — (DOCX) [file pone.0233356.s004.docx]

**Table S3 Relative abundance of species with Percentage >0.1%.**

| Species | R-F | S-F-5 | S-F-10 | S-F-30 |
| --- | --- | --- | --- | --- |
| *Bacillus aryabhattai* | 2.27±0.33A | 2.33±0.24A | 0.53±0.05B | 0.19±0.05B |
| *Rhizobium radiobacter* | 1.00±0.16A | 0.88±0.07A | 0.64±0.05A | 0.76±0.18A |
| *Bacillus drentensis* | 1.10±0.12A | 1.07±0.10A | 0.36±0.04B | 0.16±0.04B |
| *Paenarthrobacter nitroguajacolicus* | 1.08±0.15A | 0.47±0.06B | 0.48±0.04B | 0.33±0.01B |
| *Acinetobacter calcoaceticus* | 1.85±0.14A | 0.08±0.02B | 0.01±0.01B | 0.18±0.07B |
| *Bacillus simplex* | 0.22±0.02AB | 0.43±0.03A | 0.17±0.01BC | 0.14±0.02C |
| *Rhizobium etli* | 0.36±0.05A | 0.21±0.03B | 0.21±0.03B | 0.15±0.08B |
| *Rhodococcus erythropolis* | 0.03±0.01C | 0.19±0.05B | 0.31±0.04A | 0.31±0.05A |
| *Sphingobacterium multivorum* | 0.28±0.15A | 0.15±0.05AB | 0.04±0.02B | 0.09±0.01AB |
| *Solanum torvum* | 0.08±0.02B | 0.17±0.04A | 0.07±0.04B | 0.08±0.01B |
| *Stenotrophomonas rhizophila* | 0.01±0.01C | 0.20±0.02A | 0.11±0.04B | 0.07±0.02BC |

Values are presented as the mean ± standard deviation (n=3). Different letters in the same column indicate a significant difference at p < 0.01. R-F: rotation soil; S-F-5: soil of potato continuous cropping for 5 years; S-F-10: soil of potato continuous cropping for 10 years; S-F-30: soil of potato continuous cropping for 30 years.
